# Selective Homologous Expression of Recombinant Manganese Peroxidase Isozyme of Salt-Tolerant White-Rot Fungus *Phlebia* sp. MG-60, and Its Salt-Tolerance and Thermostability

**DOI:** 10.4014/jmb.2108.08042

**Published:** 2021-12-11

**Authors:** Ichiro Kamei, Nana Tomitaka, Taichi, Motoda, Yumi Yamasaki

**Affiliations:** 1 Faculty of Agriculture, University of Miyazaki, 1-1, Gakuen-kibanadai-nishi, Miyazaki 889-2192, Japan; 2 Graduate School of Agriculture and Engineering, University of Miyazaki, 1-1, Gakuen-kibanadai-nishi, Miyazaki 889-2192, Japan; 3 Faculty of Regional Innovation, University of Miyazaki, 1-1, Gakuen-kibanadai-nishi, Miyazaki 889-2192, Japan

**Keywords:** Manganese peroxidase, salt tolerance, homologous expression, white-rot fungi, *Phlebia* sp. MG-60

## Abstract

*Phlebia* sp. MG-60 is the salt-tolerant, white-rot fungus which was isolated from a mangrove forest. This fungus expresses three kinds of manganese peroxidase (MGMnP) isozymes, MGMnP1, MGMnP2 and MGMnP3 in low nitrogen medium (LNM) or LNM containing NaCl. To date, there have been no reports on the biochemical salt-tolerance of these MnP isozymes due to the difficulty of purification. In present study, we established forced expression transformants of these three types of MnP isozymes. In addition, the fact that this fungus hardly produces native MnP in a high-nitrogen medium (HNM) was used to perform isozyme-selective expression and simple purification in HNM. The resulting MGMnPs showed high tolerance for NaCl compared with the MnP of *Phanerochaete chrysosporium*. It was worth noting that high concentration of NaCl (over 200 mM to 1200 mM) can enhance the activity of MGMnP1. Additionally, MGMnP1 showed relatively high thermo tolerance compared with other isozymes. MGMnPs may have evolved to adapt to chloride-rich environments, mangrove forest.

## Introduction

White-rot fungi have a unique ability to degrade high-molecular weight aromatic polymer, lignin, via the secretion of extracellular lignin-degrading enzymes such as manganese peroxidase (MnP), lignin peroxidase, versatile peroxidase, and laccase [1-3]. MnP oxidizes Mn^2+^ to Mn^3+^ in an H_2_O_2_-dependent reaction, and Mn^3+^ organic acid chelates oxidize several lignin model compounds and synthetic lignin via the formation of a phenoxy radical [4-6]. Additionally, MnP participates in lignin biodegradation via thiol and lipid-derived free radicals that are able to oxidize a variety of nonphenolic aromatic compounds [4, 7]. Recently, there are many reports about the biodegradation of industrial pollutants by MnPs [8], specifically about their removal of hazardous wastes and bioremediation of organopollutants in water [9, 10], and bleaching and pulping of cellulose [11]. Many of these studies focused on the isolation of MnP isozyme and efficiency in the degradation of organic compounds including dyes. Commonly, MnPs are secreted in multiple isoforms in carbon- and nitrogen-limited media supplemented with Mn^2+^ and veratryl alcohol [12, 13].

The marine fungus *Phlebia* sp. strain MG-60 was isolated and selected from driftwood collected from mangrove stands in Okinawa, Japan, based on lignin biodegradability under hypersaline conditions [14]. *Phlebia* sp. strain MG-60 produces MnP mainly under hypersaline conditions [15]. It was able to brighten unbleached hardwood kraft pulp extensively even under conditions of 5% (w/v) sea salts. In contrast, pulp was only slightly brightened by the widely studied white-rot fungus *Phanerochaete chrysosporium* at 3% (w/v) and 5% (w/v) sea salt concentrations [14, 16]. The expression pattern of MnPs in nitrogen-limited cultures of *Phlebia* sp. strain MG-60 (MGMnPs) is differentially regulated under hypersaline conditions at the mRNA level [15]. When MG-60 was cultured in nitrogen-limited medium containing 3% (w/v) sea salts, higher activity of MnP was observed than that observed in normal medium. Three MnP-encoding genes (*MGmnp1*, *MGmnp2*, and *MGmnp3*) were isolated and their corresponding isozymes were identified, and enhancement of the expression of *MGmnp2* and *MGmnp3* by the addition of NaCl were reported [15]. However, the biochemical differences of each isozyme were not identified. Recently, it was reported that this fungus also has the potential of biorefinery agent caused by the potential as a biorefinery agent due to it highly selective degradation of lignin and its saccharification and simultaneous fermentation of cellulose and hemicellulose [17-20]. Since *Phlebia* sp. MG-60 is a marine white-rot fungus, it is speculated that the lignin-degrading enzyme may have high salt tolerance, although there are no reports on this.

Normally, to identify the biochemical differences between MnP isozymes, purification of each isozyme is essential. However, the difference in timing and amount of expression of each enzyme and similar molecular weight make them cumbersome to purify. Our previous paper reported that the transformation of *Phlebia* sp. MG-60 for forced expression of *MGmnp2* was done successfully and moreover the specific expression of *MGmnp2* under high-nitrogen condition (non-ligninolytic condition meaning expression of native ligninolytic enzymes are supressed) was possible [21]. This result led us to the hypothesis that forced expression of each MnP isozyme gene, specifically under non-ligninolytic condition makes it convenient to purify each MnP isozyme, compared with the purification by several chromatographic purification steps by the avoidance of contamination of native MnP isozyme.

In the present study, transformants with forced expression of *MGmnp1* and *MGmnp3* were constructed in addition to *MGmnp2*. Then, each MnP isozyme was purified simply from non-ligninolytic culture with each transformant. Finally, the biochemical traits of each simply purified MnP, such as salt-tolerance or thermostability, were investigated.

## Materials and Methods

### Fungal Strain and Incubation Condition

*Phlebia* sp. MG-60 TUFC40001 (Fungal/Mushroom Resource and Reserch Center, Japan) and its transformants were maintained on potato dextrose agar (PDA) medium. For the isolation of genomic DNA, potato dextrose broth (PDB) medium was used. For the selection of transformants showing the specific expression of each MnP isozyme, Kirk’s high-nitrogen medium (HNM) [22] was used. The forced expression transformants of *MGmnp2* were generated in our previous study [21].

### Construction of MGmnp1 and MGmnp3 Expression Vectors

The expression vectors under the control of the PbGPD (glyceraldehyde-3-phosphate dehydrogenase of *Phlebia brevispora* HHB-7030 genomic DNA; protein ID: 29450) gene promoter and terminator for *MGmnp1* (p*PbGPD*-*MGmnp1*) and *MGmnp3* (p*PbGPD*-*MGmnp3*) were constructed as described below. The PbGPD gene was obtained by PCR amplification with the primers PbGPD-F1 and PbGPD-R1 [21], and the amplified fragment was ligated into the T-Vector pMD20 (Takara Bio Inc., Shiga, Japan). The added *Asc*I restriction enzyme sites, included in the PbGPD promoter and PbGPD terminator, were obtained using the primers *PbGPD-Asc*-F1 and *PbGPD-Asc*-R1 [21]. *MGmnp1* and *MGmnp3* genes containing the added *Asc*I restriction enzyme site were amplified with PCR by using primer sets, *gMGmnp1-Asc*-F1 (GGGCGCGCCATGGCTTTCAGACAGCTTC) and *gMGmnp1-Asc*-R1 (GGGCGCGCCTATTTAGGACGGAGGGAC), *gMGmnp3-Asc*-F1 (GGGCGCGCC ATGGCCTCCAAGTTTGCT) and *gMGmnp3-Asc*-R1 (GGGCGCGCCCTAAGAGTCGTCGCCGTC), which were designed based on the *Phlebia* sp. MG-60 *MGmnp1* and *MGmnp3* gDNA sequence data (Accession numbers: AB971351 and AB971353), respectively. The amplified *MGmnp1* and *MGmnp3* genes were ligated into the expression plasmid after digestion by *Asc*I (New England Biolabs Japan Inc., Japan) according to DNA Ligation Kit Mighty Mix (Takara Bio Inc.) instructions, and then transformed into competent *Escherichia coli* JM 109 (Takara Bio Inc.) for amplification.

### Transformation

Protoplast isolation and polyethylene glycol (PEG)-mediated co-transformation assays with MG-60 were performed according to our previous study [21]. Briefly, MG-60 was pre-cultured in 100 ml CYM (yeast extract 2 g/l, polypeptone 2 g/l, glucose 20 g/l, MgSO_4_-7H_2_O 0.5 g/l, KH_2_PO_4_ 0.46 g/l, K_2H_PO_4_ 1 g/l, vitamin B1 1 mg/l, thiabendazole 20 μg/l, pH 6.0) medium for 3 days without shaking. After pre-culture, the mycelia were homogenized for 10 sec and sub-cultured for 3 days in CYM medium. The mycelia were harvested by filtration and treated with 0.5 M MgSO_4_ buffer (0.5 M MgSO_4_-7H_2_O, 20 mM maleic acid) containing 2.5% (w/v) Cellulase Onozuka (Yakult, Japan) and 2.5% (w/v) lysing enzymes from Trichoderma (Sigma-Aldrich, USA) at 30°C on a shaker (NTS-4000B) (Tokyo Rikakikai Co, Ltd., Japan) at 60 rpm for 4 h. The mycelial suspension was overlaid with 1.0 M SorbOsm (1.0 M sorbitol, 10 mM MES, pH 6.3) and centrifuged at 1,500 ×*g* in a TOMY LC-122 centrifuge (Tomy Seiko Co., Ltd., Japan) for 20 min. Protoplasts that accumulated at the interface between the two liquid phases were collected, equaling approximately 7.5×10^6^ protoplasts per one gram of wet mycelial weight. Then, 1.5×10^6^ protoplasts in 500 μl of 1.0 M SorbOsm were treated with 300 μl of DNA solution containing 20 μg plasmid in 1 M sorbitol and 0.04 M CaCl_2_. The tube was placed on ice for 30 min. After treatment, 800 μl of 50%(w/v) PEG4000 and then 75 ml regeneration medium were added to the tube. The entire contents were poured onto 50 petri dishes (2.0×104 cells/ml), and incubated at 30°C. Transformants with hygromycin resistance were selected by treatment with 15 μg/ml hygromycin B (Life Technologies, USA). M*Gmnp1* and M*Gmnp3* transformants were selected by genomic PCR amplification with the primers *PbGPD-prom*-F1 and *gMGmnp1-Asc*-R1or *gMGmnp3-Asc*-R1.

### Enzyme Activity Assay and Selection

Wild-type and isolated transformants were cultured in 10 ml HNM at pH 4.5 in 100-ml Erlenmeyer flasks. After inoculation, the cultures were incubated for 5 to 20 days at 28°C in the dark, then 1 ml of culture was collected every 5 days. Crude enzymes were prepared by filtration and centrifugation (15,000 ×*g*, 4°C, 10 min). MnP activity was determined spectrophotometrically by measuring the oxidation of 2,6-dimethoxyphenol to coerulignone (ε = 49.6 mM^−1^cm^−1^) in 50 mM malonate buffer (pH 4.5) containing 1.0 mM MnSO_4_ 1.0 mM 2,6-dimethoxyphenol and 0.2 mM H_2_O_2_ at 469 nm, 37°C. One unit of peroxidase activity was deﬁned as the amount of enzyme required to oxidize 1 μmol of 2,6-dimethoxyphenol per min.

### Preparation of Recombinant MnP Isozymes

Selected transformants showing high peroxidase activity in HNM were inoculated into 500-ml Erlenmeyer flasks with 100 ml Kirk’s HNM each, then the cultures were incubated at 28°C for production of sufficient peroxidase activity (10 to 20 days). After incubation, the mycelium was removed by the filtration with Miracloth (Merck, Germany). PEG (average molecular weight, 3,000) was added to the filtrate to make a 5% solution. The pH was then adjusted to 7.2 with 5 N aqueous NaOH. After the slime was filtered off, the filtrate was loaded onto a DEAE-Sepharose (Pharmacia) column equilibrated with 20 mM phosphate buffer (pH 7.2). The column was eluted successively with the following buffers: 20 mM phosphate (pH 6.0), 20 mM succinate (pH 5.5), and 50 mM succinate (pH 4.5). Fractions containing MnP activity were eluted with 50 mM succinate (pH 4.5). This partially purified MnP fraction was concentrated by ultrafiltration (10 kDa cut-off), then the concentrated MnP fraction was used for SDS-PAGE and enzymatic experiments. Relative activity on medium before and after purification was shown in Table 1. As control, same preparation was carried out from the Kirk’s LNM [22] with *P. chrysosporium* (PCMnP).

### Enzyme Activity under Different Salt Concentrations

NaCl was added to 71 mM malonate buffer (pH 4.5) at concentrations of 0, 250,580, 770, 1025, 1282 mM respectively. Then, 700 µl of these malonate buffers with NaCl were used for the enzyme assay by the mixing with 50 µl of 20 mM MnSO_4_, 50 µl of 20 mM 2,6-dimethoxyphenol, 100 µl of MnP enzyme and 100 µl of 2 mM H_2_O_2_. We then started and monitored the reaction of oxidation of 2,6-dimethoxyphenol according to method described before [21]. The actual concentration of NaCl in the reaction mixture was 0, 175, 406, 539, 717, and 897 mM, respectively. The remaining activities were indicated as relative value compared with the enzymatic activity without NaCl.

### Salt-Tolerance Experiment of MnP Isozyme

One hundred microliters of MnP enzyme solution was mixed with 700 µl of 71 mM malonate buffer (pH 4.5) with NaCl at concentrations of 0, 250,580, 770, 1025, and 1282 mM respectively in test tube (the actual concentrations of NaCl in test tubes are 0, 219,508, 674, 897, and 1122 mM respectively) and incubated for 30 or 120 min at 37°C in the dark. Then, 50 µl of 20 mM MnSO_4_, 50 µl of 20 mM 2,6-dimethoxyphenol, and 100 µl of 2 mM H_2_O_2_ were added and the reaction of oxidation of 2,6-dimethoxyphenol was monitored according to method described before [21]. The remaining activities were indicated as relative value compared with the enzymatic activity as measured in the buffer with NaCl immediately without incubation in malonate buffer with NaCl.

### Thermo Tolerance of MnP Isozymes

One hundred microliters of MnP enzyme solution was incubated at different temperatures (4, 20, 25, 30, 35, 40, 45, 50, 60, and 70°C) for 60 min. Immediately afterwards, the enzyme was immersed in an ice bath and then enzyme was mixed with 700 µl of these malonate buffers, 50 µl of 20 mM MnSO_4_, 50 µl of 20 mM 2,6-dimethoxyphenol, and 100 µl of 2 mM H_2_O_2_, and then we started and monitored the reaction of oxidation of 2,6-dimethoxyphenol according to method described before [21]. The remaining activities were indicated as relative value compared with the enzymatic activity after incubation at 4°C.

## Results and Discussion

### Selection of Transformants

One hundred randomly isolated strains with hygromycin resistance were selected by genomic PCR amplification with the primers *PbGPD-prom*-F1 and *gMGmnp1-Asc*-R1 or *gMGmnp3-Asc*-R1 to confirm the insertion of p*PbGPD*-*MGmnp1* and p*PbGPD*-*MGmnp3*, respectively. The insertion efficiency (co-transformation efficiency) of p*PbGPD*-*MGmnp1* was 77% (77 co-transformants/100 transformants with hygromycin resistance) and that of p*PbGPD*-*MGmnp3* was 62% (62 co-transformants/100 transformants with hygromycin resistance). Our previous study reported that the co-transformation efficiency of MGmnp2 expression vector (pPbGPD-MGmnp2) was 89.1% (181 co-transformants/203 transformants with hygromycin resistance) [21]. It was also reported that the resulting efficiency of co-transformation of p*PbGPD*-HPT and knockdown construct of pyruvate decarboxylase gene (pMGPDC-RNAi) was 75% (108 co-transformants/144 hygromycin-resistant strains) [23]. From these high efficiency co-transformation results, it was concluded that the co-transformation strategy is effective for the transformation of *Phlebia* sp. MG-60.

**Fig. 1:**
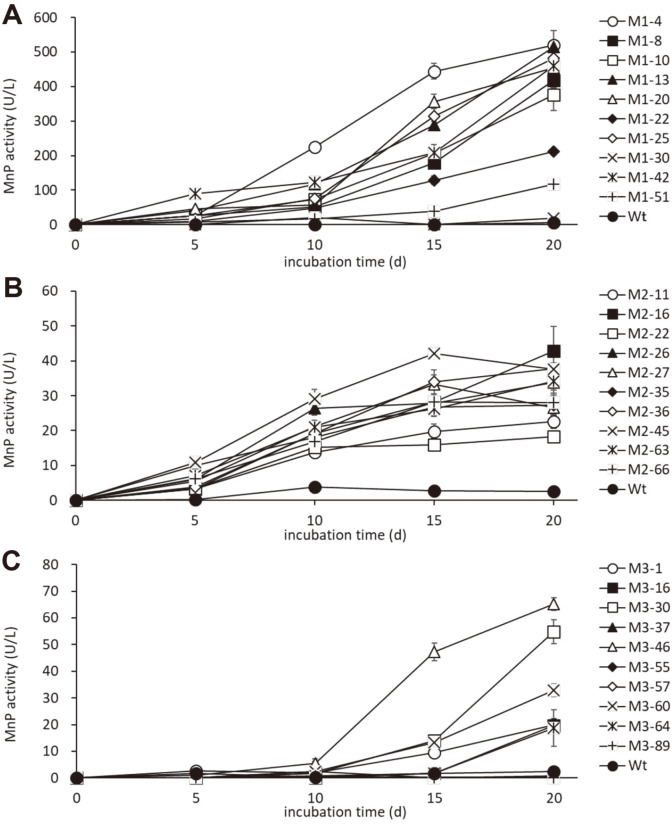
Production of MnP activity by the forced expression of *MGmnp1* (**A**), *MGmnp2* (**B**) and *MGmnp3* (**C**) in HNM. Wt: native *Phlebia* sp. MG-60.

Ten transformants were selected from the co-transformants with each MnP expression vector and we then checked the MnP activity in Kirk’s-HNM. Fig. 1 showed the accumulation of MnP activity during 20 days' incubation in 10 ml of HNM. Although a trace amount of MnP activity was observed in the culture with wild-type *Phlebia* sp. MG-60, the transformant line of p*PbGPD*-*MGmnp1*showed significant accumulation of MnP activity except for the transformant line MnP1-30. The highest accumulation of MnP activity was observed in the transformant line MnP1-4. In the case of p*PbGPD*-*MGmnp3* transformants, lines MnP3-1, -16, -30, -46, -60 and -64 showed higher accumulation of MnP activity compared with the trace activity of wild type. The highest accumulation of MnP activity was observed in the culture with MnP3-46. In the case of p*PbGPD*-*MGmnp2* transformants, all co-transformants showed higher MnP activity accumulation than wild type and the highest accumulation was observed in the culture with MnP2-45. It was reported that the addition of NH_4_^+^ (HNM) could supress the production of MnP activity in the culture with *Phlebia* sp. MG-60 [21,24], and that the addition of NH_4_^+^ could delay the appearance of ligninolytic activity in *P. chrysosporium* [25]. In the present study, MnP activity was significantly suppressed in the HNM culture and transformants which was transformed with MGMnP isozyme genes and showed significant accumulation of MnP activity. Therefore, these MnP activities suggest that the specific expression of each recombinant MnP isozyme was done successfully.

Relatively higher activities of MnP were observed in the culture with the transformants of p*PbGPD*-*MGmnp1* (maximum is approx. 500 U/L) compared with p*PbGPD*- *MGmnp2* (maximum is approx. 50 U/L) or p*PbGPD*-*MGmnp3* (maximum is approx. 70 U/L) although the common GDP promoter dehydrogenase in *P. brevispora* HHB-7030 was used for expression. It was reported that the expression of *MGmnp1* is dominant in LNM without salt, however, *MGmnp2* and *MGmnp3* expressed in the culture with sea salts or NaCl dominantly [15]. It it was also reported that the transcription level of *MGmnp2* did not collate with MnP activity [15]. From these experimental results, therefore, involvement of post-transcriptional regulation was also suggested. According to the general eukaryotic rule for N glycosylation (Asn-X-Ser/Thr) [26], two and three asparagines potentially involved in glycan linkage were found in MGMnP2 (N132 and N218) and MGMnP3 (N132, N218, and N348), whereas only one (N103) was found in MGMnP1 [15]. Different numbers of glycosylation positions may affect these expression traits.

The time-dependent production of MnP activity is also different between wild type in Kirk’s-LNM and transformant in Kirk-HNM. In previous studies, wild-type MnP activity of *Phlebia* sp. MG-60 in LNM showed a short peak at 6-10 days and then decreased rapidly [15]. On the other hand, in the present study, transformants accumulated MnP activity in HNM in a time-dependent manner until day 20. This trait suggested that the production of MnP activity in the LNM by the wild type depends on the transient gene transcription, and there are some factors responsible for the rapid inactivation of the enzyme once made. The ability to accumulate each MnP isozyme in a time-dependent manner in the present study may be an advantage in obtaining an isozyme to investigate various properties of the enzyme.

### Easy Purification of MnP Isozymes

**Fig. 2:**
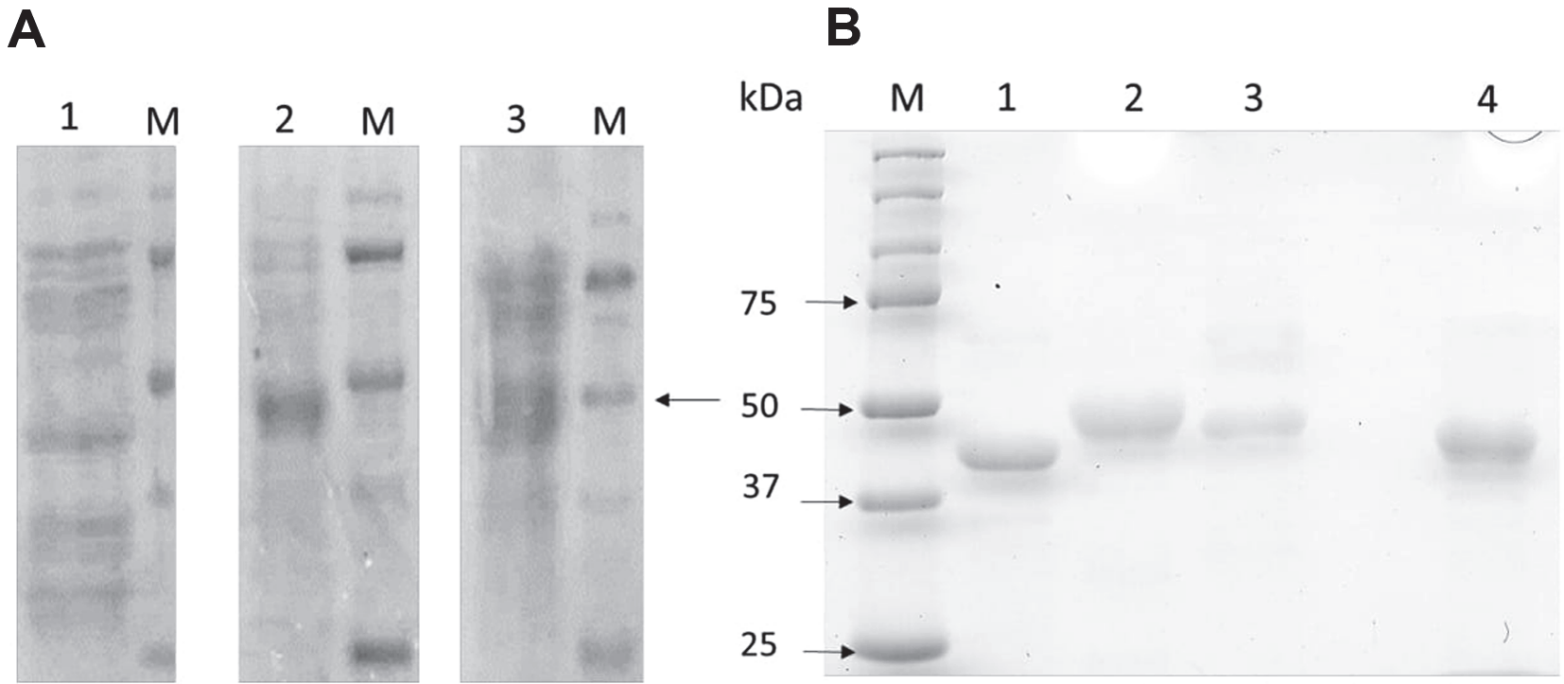
SDS-PAGE of the non-purified (**A**) and purified (**B**) MnPs. MGMnP1 (Lane 1), MG-MnP2 (Lane 2), and MGMnP3 (Lane 3), M: protein markers, Lane 4: MnP from *P. chrysosporium*.

Transformant lines MnP1-4, MnP2-46 and MnP3-45 were selected based on the productivity of MnP activity in HN medium as described above. Then, each transformant was cultured in 100 ml of HNM and the separated culture both was loaded and easily purified by DEAE-Sepharose, then the fraction eluted with 50 mM succinate (pH 4.5) was collected and analyzed. Fig. 2A showed SDS-PAGE analysis of the obtained enzyme fraction before purification. Although a trace amount of protein remained at near 75 kDa, each enzyme solution of the transformant lines MnP1-4, MnP2-46 and MnP3-45 showed a strong single band on SDS-PAGE level at around 45, 49, and 47 kDa, respectively (Fig. 2B). In our previous study, the corresponding isozymes of the MnP isozyme genes *MGmnp1*, *MGmnp2*, and *MGmnp3* were identified by peptide mass fingerprinting analysis as the MnP isozymes at 45, 50, and 47 kDa respectively. The size order of each enzyme was the same as in previous study, but the molecular weight of MGMnP2 seemed a little low. This may have been caused by failed glycosylation. As mentioned above, MGMnP2 has two asparagines potentially involved in glycan linkage (N132 and N218) [15]. However, overall, selective MGMnPs expression and easy purification of recombinant MnP isozymes in HNM was successfully done. Recombinant MnP expression has been reported in *E. coli*, however, in vitro has refolding would be a way to obtain active protein, and then low yields and the lack of glycosylation should be overcome [27, 28]. Therefore, the selective expression of the recombinant MnP isozymes in HNM in the present study may be applicable to MnP with different origin, and may be used as an applied expression system.

### Effect of the Salt on the Activity and Salt-Tolerance of MnPs

**Fig. 3:**
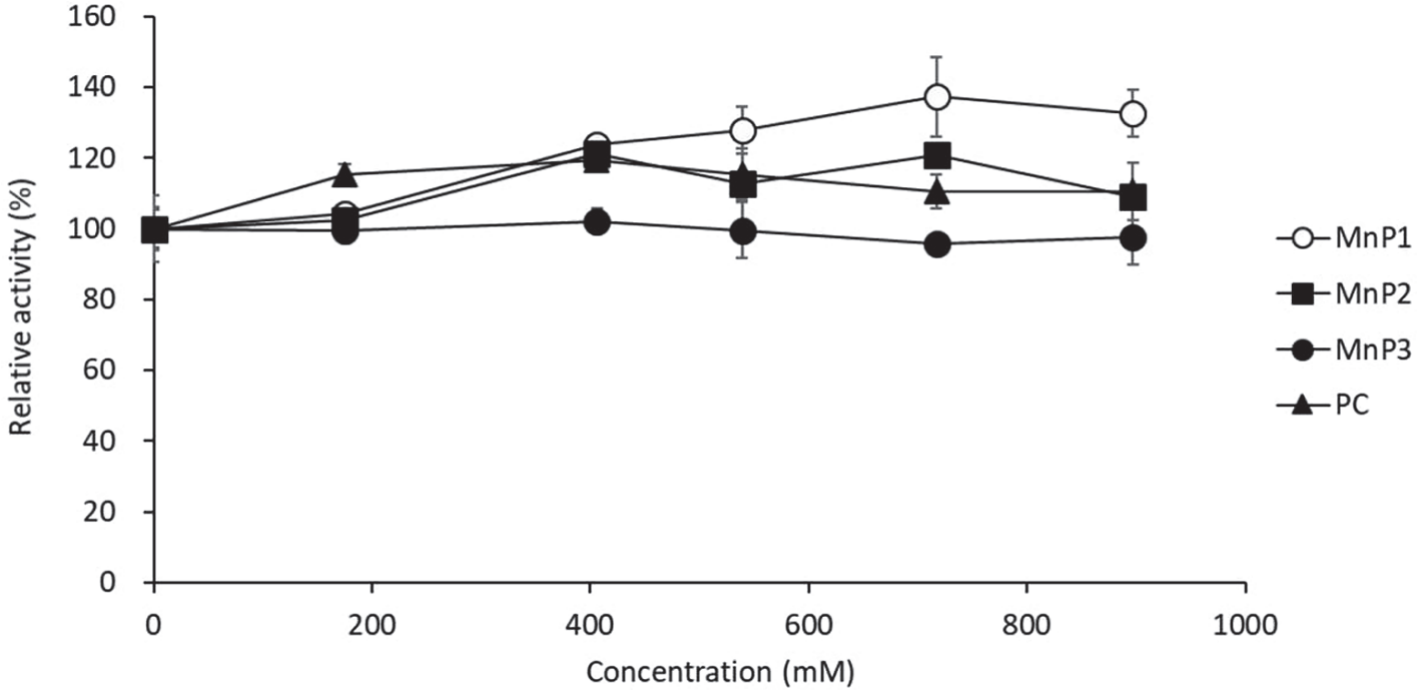
Effect of the NaCl on MGMnP activity. Activities of each purified MnPs were measured in the reaction mixture with different concentrations of NaCl. The remaining activities were indicated as relative value compared with the enzymatic activity without NaCl (Concentration = 0 mM).

To test the saline tolerance, enzyme activity was determined under different NaCl concentration. Fig. 3 showed the enzyme activity of each recombinant MGMnP under different NaCl concentration. Relative activity compared with 0 mM NaCl showed no decrease of activity in the reaction mixture with NaCl. Interestingly, enzyme activity of MnP1 tended to increase in the reaction mixture containing high concentration of NaCl. Si and Cui reported that the activity of MnP from *Perenniporia subacida* (Pspd) was stimulated by chloride ion, however, high concentration of NaCl decreased the activity of Pspd slightly [29]. Therefore, the result that the activity of MGMnP1 is enhanced by high concentration of NaCl may be a biochemical feature in *Phlebia* sp. MG-60, and MGMnPs may have better salt-tolerance compared with Pspd.

**Fig. 4:**
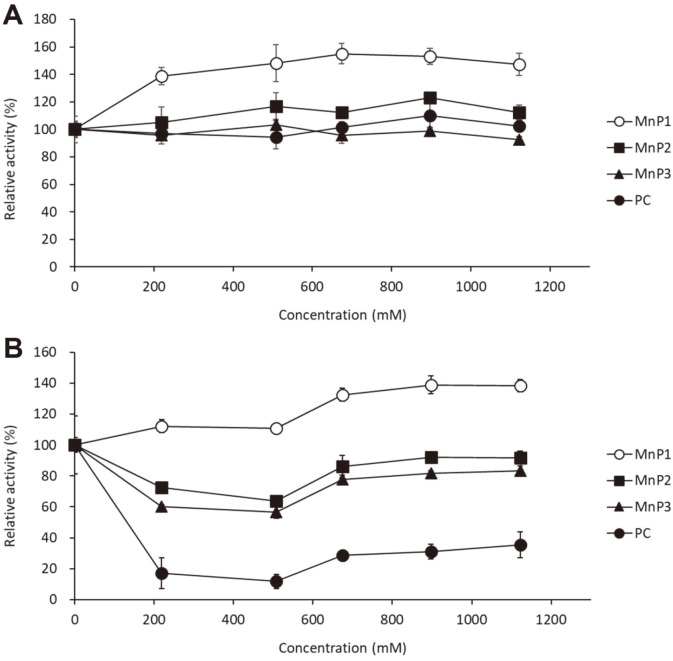
Salt-tolerance of MGMnPs. Each purified MnP was pre-incubated with different concentration of NaCl for 30 min (**A**) or 120 min (**B**) then the activity was measured. The remaining activities were indicated as relative value compared with the enzymatic activity at measured in the buffer with NaCl immediately without incubation in malonate buffer with NaCl (Concentration = 0 mM). PC means MnP from *P. chrysosporium*.

The activity was measured immediately after addition of buffer containing NaCl to determine the effect of NaCl on enzyme activity in the above experiment. However, the activity tolerance under high salt concentration condition has not yet been investigated. In order to estimate the tolerance of each MGMnP toward NaCl, the residual activity when stored at different salt concentrations for 30 min and 2 h was examined (Fig. 4). After 30 min stored at 37°C, there was no decrease in the activity of each MnP by incubation with NaCl-containing buffer. Furthermore, the activity of MnP1 tended to be stimulated by the incubation for 30 min with NaCl-containing buffer compared with the unincubated enzyme. After 2 h incubation at 37°C, the activity of PCMnP was decreased drastically up to 10 to 20% remaining compared with unincubation. On the other hand, the decrease in activity of MGMnP2 and MGMnP3 (50 to 80% remaining) was milder than that of PCMnP, and interestingly, no decrease in activity was observed for MGMnP1. These results suggest that the MnP isozymes secreted by *Phlebia* sp. MG-60 retains relatively high salt tolerance, and in particularly, MGMnP1 showed the ability to retain remarkably strong salt tolerance.

Several papers reported the effect of metallic ion for purified MnP derived from different white-rot fungi [30-32]. Overall, from previous reports, Na^+^ tends to not affect or slightly affect the activity for purified MnPs, although Ca^2+^ and Fe^2+^ tend to inhibit the activity at low concentration (1-10 mM). While it has been reported that chloride ion does not affect MnP activity derived from *P. chrysosporium*, even when present at a concentration of 250 mM [33], a low concentration of chloride ion (around 100 mM) promotes the activity of MnP derived from Pspd and a high concentration of chloride ion (over 500 mM) has been reported to reduce the activity of Pspd [29]. In the present study, it was shown that high concentration of NaCl solution had no effect on the activity and promoted the activity of MGMnP1. MGMnP2 and MGMnP3 showed relatively high tolerance for high concentration of NaCl. Since *Phlebia* sp. MG-60 is a salt-tolerant basidiomycete isolated from mangroves forest, MGMnPs may also have evolved to adapt to chloride-rich environments.

### Thermostability of *MnPs*

**Fig. 5:**
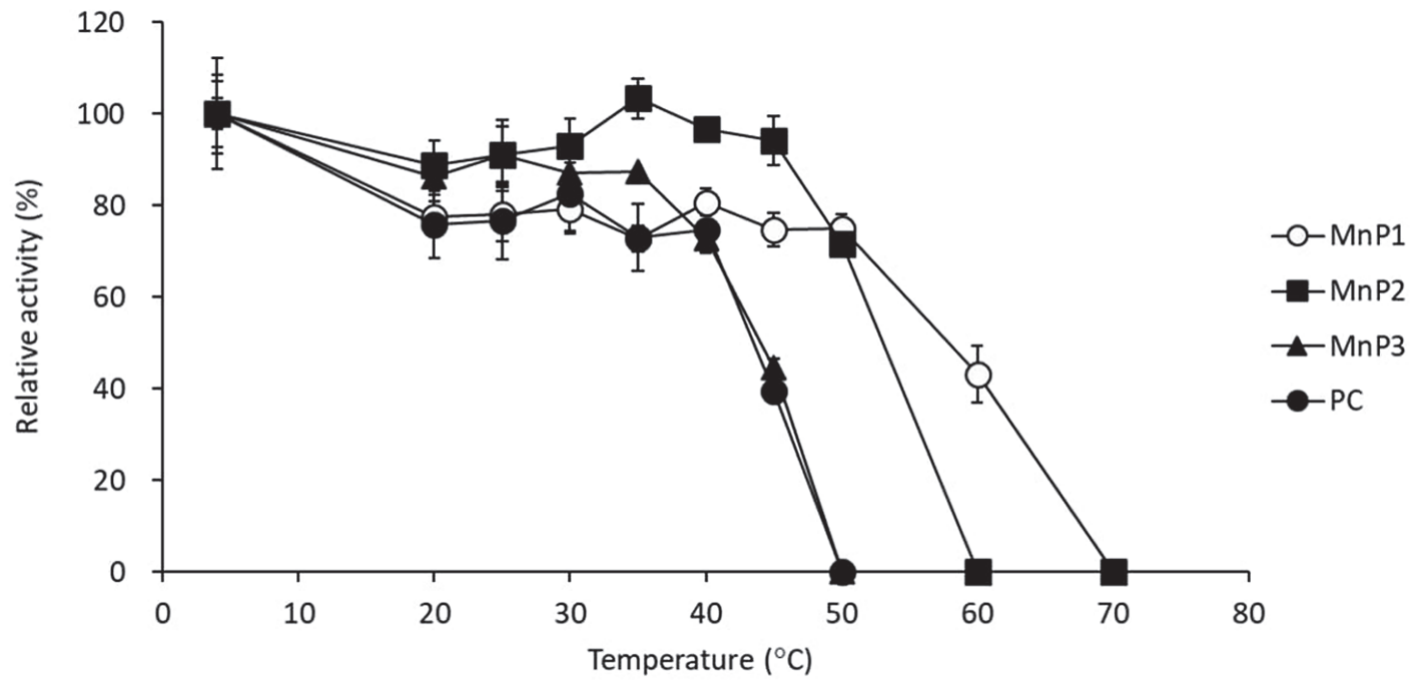
Thermostability of MGMnPs. Each purified MnP was pre-incubated with different temperature for 60 min then the activity was measured. The remaining activities were indicated as relative value compared with the enzymatic activity after incubation at 4°C. PC means MnP from *P. chrysosporium*.

Each enzyme was kept at different temperatures for 60 min and the remaining activity was examined to estimate the thermostability of each MnP enzyme by comparison with that kept at 4°C. Fig. 5 showed the remaining activity after heat treatment for 60 min. All MnP enzymes were stable up to 40°C. MGMnP3 and PCMnP showed decrease of activity over 40°C, then the activity disappeared at 50°C. MGMnP2 is stable at 45°C while the activity decreases around 50°C and disappears completely at 60°C. On the other hand, MGMnP1 keeps half of its activity at 60°C, then the activity completely disappears at 70°C. MGMnP1 has relatively high thermostability in MGMnPs. In other studies, the thermostability of purified MnPs also differed widely; it was reported that purified MnP from immobilized *P. chrysosporium* was stable at 40°C, then it was inactivated totally at 65°C [33]. MnP from *Stereum ostrea* was stable up to 35°C, but was inactivated rapidly at temperature higher and then this, then was inactivated totally at 65°C [31]. Recently, purified MnP from *Bjerkandera adusta* showed higher tolerance for temperature [34]. Compared with these reports, MGMnP1 showed relatively high tolerance for high temperature. The mangrove from which *Phlebia* sp. MG-60 was isolated is a salt marsh in tropical and subtropical regions. It may have been adapted to relatively high-temperature environments. From the phylogenetic analysis of Class II fungal secreted heme peroxidase, it was reported that MGMnP2 and MGMnP3 are contained in the group of the typical fungal Mn2-oxidizing, long MnPs. On the other hand, MGMnP1 was classified into the group of short MnPs [3]. This classification based on the amino acid sequence may also be involved in the finding that MGMnP1 has higher salt tolerance and thermostability.

In the present study, we established forced expression transformants of three types of MnP isozymes. In addition, the fact that this fungus hardly produces native MnP in a HNM was used to perform isozyme-selective expression and simple purification in HNM. This system gives a single band of each MGMnP isozyme via easy DEAE-sepharose purification, so this process showed potential to obtain each of the MnP isozymes easily. The resulting MGMnPs showed high tolerance for NaCl compared with the MnP of *P. chrysosporium*. It was worth noting that high concentration of NaCl (over 200 mM to 1200 mM) can enhance the activity of MGMnP1. Additionally, MGMnP1 showed relatively high thermostability compared with other isozymes, and it is possible that MGMnPs may have evolved to adapt to chloride-rich environments, such as mangrove forest.

## Figures and Tables

**Table 1:** Easy purification of MnP from the culture with isozyme-selective expression.

Enzyme	Purification stage	Volume(ml)	Protein amount (mg)	Total activity (U)	Relative activity (U/mg)	Recovery (%)
MnP1	Medium	80	7.85	30.44	3.88	100
	DEAE	24	1.88	14.64	7.77	48
MnP2	Medium	100	17.86	129.43	7.25	100
	DEAE	19	0.89	11.26	12.59	9
MnP3	Medium	380	31.13	78.60	2.53	100
	DEAE	14	0.87	11.29	12.99	14

Medium: Medium after removal of mycelium.

DEAE: Enzyme solution concentrated by ultrafiltration after DEAE fractionation.
